# A Delphi Consensus Approach for the Management of Chronic Pain during and after the COVID-19 Era

**DOI:** 10.3390/ijerph182413372

**Published:** 2021-12-19

**Authors:** Marco Cascella, Luca Miceli, Francesco Cutugno, Giorgio Di Lorenzo, Alessandro Morabito, Alfonso Oriente, Giuseppe Massazza, Alberto Magni, Franco Marinangeli, Arturo Cuomo, on behalf of the DELPHI Panel

**Affiliations:** 1 Division of Anesthesia and Pain Medicine, Istituto Nazionale dei Tumori, IRCCS Fondazione G. Pascale, 80100 Napoli, Italy; a.cuomo@istitutotumori.na.it; 2 Department of Electrical Engineering and Information Technologies, Università di Napoli “Federico II”, 80100 Napoli, Italy; f.cutugno@unina.it; 3 Department of Pain Medicine, IRCCS C.R.O. National Cancer Institute of Aviano, 33081 Aviano, Italy; luca.miceli@cro.it; 4 Department of Systems Medicine, University of Rome Tor Vergata, 00133 Rome, Italy; di.lorenzo@med.uniroma2.it; 5 Psychiatry and Clinical Psychology Unit, Fondazione Policlinico Tor Vergata, 00133 Rome, Italy; 6 Thoracic Medical Oncology, Istituto Nazionale Tumori, IRCCS “Fondazione G. Pascale”, 80100 Napoli, Italy; a.morabito@istitutotumori.na.it; 7 Rheumatology Unit, Department of Clinical Medicine and Surgery, University Federico II, 80100 Napoli, Italy; prof.aoriente@gmail.com; 8 Division of Physical Medicine and Rehabilitation, Department of Surgical Sciences, University di Torino, 10121 Torino, Italy; giuseppe.massazza@gmail.com; 9 Italian College of General Practitioners and Primary Care (SIMG), Via Del Sansovino 179, 50142 Firenze, Italy; alberto.magni@icloud.com; 10 Department of Life, Health & Environmental Sciences, University of l’Aquila, 67100 l’Aquila, Italy; francomarinangeli@gmail.com

**Keywords:** COVID-19, Delphi survey, chronic pain, telemedicine, recommendations

## Abstract

Due to a lack of published evidence on the topic, a modified Delphi approach was used to develop recommendations useful for chronic pain management during and after the COVID-19 pandemic. Focusing on the available literature and personal clinical expertise, an Italian board of nine professionals from different disciplines identified four main topics: prevention of chronic pain, treatment of chronic pain, consequences of inadequate treatment, and perspectives. They elaborated a semi-structured questionnaire. A multidisciplinary panel of experts in the field of pain management was requested to comment on the statements. Based on the answers provided, a structured questionnaire was prepared (Round 1). It included 21 statements divided into three categories (organizational issues; diagnosis and therapies; telemedicine and future perspectives). A five-point Likert scale was adopted. The threshold for consensus was set at a minimum of 70% of the number of respondents (level of agreement ≥ 4, Agree or Strongly Agree). A final questionnaire with rephrasing of the statements that did not reach the consensus threshold was elaborated (Round 2). A total of 29 clinicians were included in the panel. Twenty clinicians (69%) responded in both the first and second round. After two rounds, consensus (≥70%) was achieved in 20 out of 21 statements. The lack of consensus was recorded for the statement regarding the management of post-COVID pain (55%; Median 4; IQR 2.3). Another statement on telemedicine reached the threshold in the first round (70%), but the value was not confirmed in Round 2 (65%; Median 4; IQR 2). Most of the proposed items reached consensus, suggesting the need to make organizational changes, the structuring of careful diagnostic and therapeutic pathways, and the application of new technologies in pain medicine. Long-COVID-19 care is an issue that needs further research. Remote assistance for chronic pain must be regulated.

## 1. Introduction

Primary and secondary chronic pain conditions are included among the main reasons for seeking medical care [1]. Notably, about 19% of the adult population in Europe suffers from chronic pain [2], and more than 120 million adults in the United States are affected by chronic pain such as headaches, back and neck pain, and orofacial pain [1]. In addition, in oncology patients, pain is one of the most frequent and relevant symptoms with an incidence of up to 50% and reaches 90% in the advanced stages of the disease [3].

The COVID-19 pandemic has reduced allocated resources in the treatment of diseases other than COVID-19 [4]. In the context of care gaps for patients suffering from chronic diseases, the effects of the pandemic also reverberate far beyond patients suffering from chronic pain. Therefore, deleterious phenomena of undertreatment of pain can induce important repercussions for both patients and the healthcare system. Furthermore, worsening of chronic pain induced by the deterioration of pre-existing factors, and unmasking newly triggered cases due to risk factors including psychosocial stressors or organ-specific biological causes, are worrying issues that may require an increase in healthcare needs after the COVID-19 crisis [5]. Previously, at the beginning of the pandemic, an expert panel composed of pain specialists, psychologists, and researchers formulated a set of recommendations to guide the management of patients with pain [6]. During the crisis, the scenarios changed rapidly. Consequently, it is difficult to refer to strict criteria. Furthermore, it is necessary to start looking to the future, planning new strategies useful in the post-pandemic era.

Based on these premises, it is desirable to have a clear picture of the problem, to define the most effective measures to reduce the consequences of undertreatment for chronic pain, and to evaluate the opportunities that the pandemic could offer. Due to the insufficient knowledge in this field, a modified Delphi approach was used to develop a set of recommendations useful for the management of chronic pain conditions during and after the COVID-19 era.

## 2. Methods

### 2.1. Study Design

**Figure 1:**
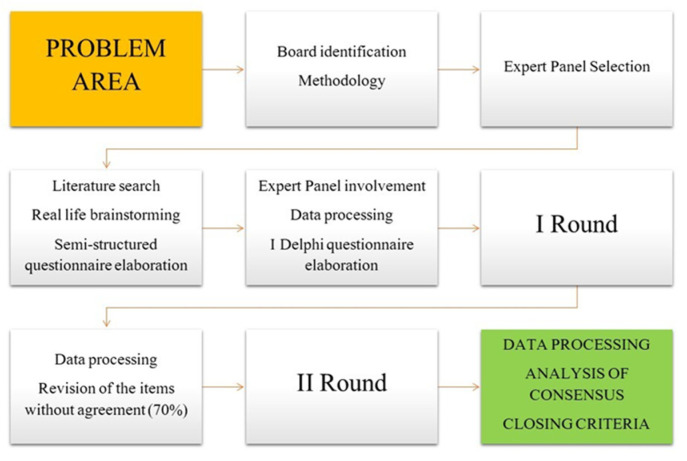
Flow chart of the modified Delphi survey.

A two-round modified Delphi method was used. The Delphi approach provides a group consensus strategy that, through the literature review, the opinions of stakeholders, and expert judgments aims to obtain agreement within a field [7]. After the identification of the research problem, a thorough literature search was conducted, and a semi-structured questionnaire was developed. Later, a multiprofessional group of experts in pain management received an e-mail invitation to participate in the study and complete questionnaire rounds. A member of the board (M.C.) was the moderator. The board did not adopt the stability criterion because it can be confusing and is rarely used as a closing criterion [8]. Ultimately, two rounds were decided a priori as closing criterion (Figure 1). 

### 2.2. Study Participants

Two types of participants were involved in the Delphi process: members of the expert committee (the board) and members of the panel of experts (the panel). The panelists were selected by the expert committee. Selection criteria included:

- Being active pain specialists.
- General practitioners who usually manage at least 25 patients/year with chronic pain.
- Professionals from different disciplines (pain therapy, oncology, general medicine, rheumatology, neurology, geriatrics, pediatrics, orthopedics, and physiatry) with a documented experience in the treatment of chronic pain.

The participation in scientific boards on pain, affiliation with scientific societies, a scientific activity characterized by at least three publications about pain in the last 10 years, and being a member of the editorial board of scientific journals were adopted as additional selection requirements. Two members of the board (L.M. and A.M.) and the moderator verified the congruence of the requirements. Upon acceptance for participation in the study, each panelist confirmed the eligibility criteria.

In every round, anonymous electronic surveys were used to collect the data via Google Form in its free release. Non-responders were sent two electronic reminders.

### 2.3. Questionnaire Design Process

Based on the literature review and clinical experience, board committees defined the topics, and an online meeting was held in December 2020. The topics were the lack of prevention for chronic pain, the gaps in pain treatment and their consequences, and perspectives for addressing pain management during and after the pandemic. The participants strongly emphasized that the most important issues were the entity, severity, and complexity of healthcare needs. Undoubtedly, these gaps must be addressed through therapeutic planning and advanced resources such as tools for remote monitoring/visits. Thus, the expert committee reviewed the topics and defined a semi-structured questionnaire containing 16 items across 4 topic areas as follows (Table 1):

- Prevention of chronic pain (1 item).
- Chronic pain treatment (7 items).
- Consequences (3 items).
- Perspectives (5 items).

In each item, three statements have been inserted to guide in the expression of comments. Due to the health emergency and the need to intervene as soon as possible, we asked the participants to respond quickly.

Based on the considerations expressed by the panel in the notes section, the moderator, two members of the Board (A.C., and F.M.), and the linguistic and methodology expert (F.C.) prepared a structured questionnaire. It included 18 closed statements divided into three categories:

- Organizational issues (8 items).
- Diagnosis and therapy (6 items).
- Telemedicine and future perspectives (7 items).

The questionnaire was submitted to the whole board for approval before administration to the panel. The first Delphi round was then launched. Panelists used a dedicated online platform, and a timeline of 7 calendar days to answer was established. A further 3 days were granted after a reminder e-mail. 

About the agreement, a 5-point Likert scale (1: strongly disagree; 2: disagree; 3: partially agree; 4: agree; 5: strongly agree) was adopted. The threshold for consensus was set at a minimum of 70% of the number of respondents with a level of agreement ≥ 4 (Agree or Strongly Agree). Statements that reached the pre-established cut-off were directly included in the final recommendations. 

For the statements that had not reached the cut-off, a revaluation (rephrase) was carried out. Then, the second Delphi round was started. It provided the same rules as the first. Finally, analysis of consensus and data processing were performed, and a closing meeting was held.

## 3. Results

A Delphi-based survey consisting of a preliminary round (semi-structured questionnaire) and two subsequent rounds (First Delphi round and Second Delphi round) was conducted between May and September 2021. A total of 29 clinicians were included in the Panel. Respondents were 20 (69%) both in the first round and in the second one (Table 2).

After the first Delphi round, 16/21 statements reached the cut-off. Based on the responses and comments, the board reformulated the five statements that had not reached the set threshold. Finally, in the second round, consensus (≥70%) was achieved for 20 out of 21 statements. The lack of consensus was recorded for the statement regarding the management of post-COVID pain. In this statement, a score ≥ 3 was obtained in 75% of panelists (Median 4; IQR 2.3). Another statement on telemedicine reached the threshold in the first round (70%), but the value was not confirmed in the second consultation (65%; Median 4; IQR 2). (Table 3)

## 4. Discussion

After the second Delphi round, all but one of the statements reached the prefixed cut-off. Another statement reached the threshold in the first round, but the value was not confirmed in the second consultation. About the first block of organizational problems, the recommendations concern the need to extensively reshape the organization of chronic pain therapy. Careful therapeutic pathways must be activated also through the application of priority criteria, for example, to program interventional and surgical procedures. Moreover, both during and especially post-pandemic, the organization must foresee the planning of close control visits to reduce dangerous therapeutic delays. It must be emphasized that these pathways must be set up to simplify the processes of prescription and dispensing of drugs. In Italy, during the first wave of the pandemic (March 2020), there was a serious concern about the availability of drugs. About certain categories of medications, such as opioids, even in the context of the COVID emergency, their prescription must follow a careful case-by-case analysis, especially in the setting of non-cancer patients [9,10,11]. On the other hand, thoughtful processes must guide clinicians to avoid the risk of depriving patients of a valid therapeutic alternative [12].

A key recommendation is the need to structure personalized pathways with the involvement of the general practitioner who must act as the first filter; subsequently, they can refer complex patients to the various specialists according to well-defined multidisciplinary pathways. In the context of multi-professional approaches, it is advisable to consider activating the priority consultation with the psychologist/psychiatrist. As Goesling et al. [13] affirmed, “Psychiatrists are an important piece of the pain management puzzle”. Furthermore, ad hoc courses must be defined for pediatric patients.

The diagnostic and therapeutic approach to chronic pain concerns aspects of primary importance. When the availability of diagnostic procedures is limited, patients should be selected according to priority criteria. However, pending the completion of the instrumental diagnosis, it is advisable to start a pharmacological strategy, prescribing analgesic therapy for up to 30 days. This bridging approach also concerns the expectation for interventional or surgical therapies [14]. In this way, a dangerous interruption of therapies should be avoided, as patients could experience worsening of their clinical conditions and quality of life with a rapid progression toward disability. 

Therapeutic pathways differ according to the type of chronic pain. According to the International Association for the Study of Pain (IASP), chronic pain is a complex multidimensional experience severely compromising the quality of life, often limiting the ability to work and sleep and affecting social interactions with friends and family [15]. Consequently, the term chronic pain encompasses a multitude of clinical conditions. In the case of difficult or complex pain, it is a priority to apply protocols coordinated by the pain therapist that include multimodal therapy.

Chronic pain conditions, mostly expressed as myalgia or joint pain, are encompassed among the COVID-19 sequelae [16]. Since proper management of these patients represents an open issue, this survey addresses the topic. Nevertheless, after the first round, the statement “In patients with post-COVID-19 chronic pain it is important to provide specific therapeutic pathways” did not reach consensus. The board rephrased the sentence as “In patients with post-COVID-19 chronic pain it would be desirable to provide specific therapeutic pathways in dedicated clinics”, underlining the need to create clinics dedicated to the management of the problem. However, even in this case, the minimum expected consensus was not obtained (55%). In the Italian context, during the second pandemic phase, several regions had suggested the opening of clinics dedicated to chronic post-COVID pain. Nonetheless, this strategy seems to be not feasible in the national territory, especially in some areas where the provision of care is more inadequate. Furthermore, since the patient suffering from post-COVID pain is a patient with chronic pain, ad hoc pathways requiring considerable resources are probably unnecessary. In other words, the problem can be managed according to healthcare strategies and policies commonly adopted to manage chronic pain problems. It can be suggested that to facilitate the care of these patients, the clinical spectrum of chronic post-COVID pain could be included among the priority criteria to perform the first visit as soon as possible. Another practical suggestion is the planning of telehealth-based multidisciplinary pain management approaches [17].

Concerning the third category, several important recommendations have emerged. In the field of pain medicine, the pandemic has promoted attempts to provide patients with adequate care despite restrictions to in-presence activities [18]. Compared with the traditional in-person hospital or ambulatory visits, telemedicine can improve access to care and facilitate continuity of care. Remarkably, this approach can help optimize resources and contain high health care costs [19]. Previous investigations proved that telehealth-based programs are useful for the management of chronic cancer [20] and non-cancer pain [21]. Although attempts to release practice guidelines and recommendations for telemedicine in pain management have recently been proposed [18,22], the matter must be properly regulated. Because of the pandemic, telemedicine will probably become increasingly important for the management of patients with chronic pain. This approach could deliver tailored strategies, providing improved access to health services and creating and maintaining a therapeutic alliance in the long term. Nevertheless, several issues must be faced. They include accurate diagnosis and assessment, monitoring, and strategies useful to modify treatment, as well as privacy regulation and data storage. Additionally, to properly use the new technology, it is necessary to invest in training directed to healthcare professionals but also patients and caregivers. In our survey, the ad hoc statement reached 100% consensus in the two highest levels of agreement. Regarding the operating procedures, a large part of the panel stated that the first visit should be made in person and that remote assistance must be governed by precise rules. Consequently, the implementation of new web-based systems for the management of chronic pain requires further evaluation and well-structured pathways.

Although after the first consultation the statement “In situations of reduced availability of hours of analgesic therapy, it is advisable to implement remote care strategies for the management of primary and secondary chronic pain (including cancer pain)” reached the cut-off, it was not confirmed in the second consultation (65%). The percentage difference is minimal, and the statement was probably not well formulated by the board. Additionally, in both rounds, the median was high, indicating a positive opinion rather than a disagreement. However, these data suggest that remote assistance processes need to be structured and regulated. It is necessary, for example, to distinguish between the different problems (e.g., cancer and non-cancer pain, age groups, etc.) and provide that, for some conditions, an initial clinical evaluation should be done in person.

### Strengths and Limitations

Our study has several strengths. Our expert panel consisted of experts in different fields of medicine who have experience treating different clinical expressions of chronic pain. The Delphi methodology, and its modifications, provide for the active participation of professionals involved in the analysis process of a clinical pathway that is often difficult and where scientific evidence is lacking. The sharing of "peer" opinions represents, in fact, a recognized method of scientific advancement also based on clinical experience. Furthermore, in the building of the panel, the board included professionals who manage pain even in particular areas, such as those involving fragile patients with neurocognitive disorders, children, and elderly. Moreover, maintaining anonymity throughout the entire process was of fundamental importance for avoiding biases such as individual dominance, group pressure, and opinion conformity (groupthink) observed with face-to-face meetings.

The study was limited by its small sample size. However, there is no gold standard of sample size for Delphi panels. In published studies, the size of panel members may vary from 10 to 1000. In addition, the board has set very strict inclusion criteria. According to Powell, the success of an investigation using the Delphi approach results from the “qualification of the experts” [23]. Moreover, several Delphi studies have been conducted on less than 15 panelists [24]. Furthermore, the strict eligibility criteria led to the inclusion of high-profile professionals.

Another limitation is the inclusion of only Italian experts, both in the board and in the panel. Local policies have greatly influenced therapeutic choices during the pandemic. The choice of quarantine measures, vaccines, and surveillance were different between countries. Under these premises, it would have been difficult to standardize consensus on operational strategies that were conditioned by local and well-contextualized choices. Once the pandemic has been overcome, the board suggests structuring a further qualitative investigation process that may involve experts worldwide. This new survey will focus primarily on post-pandemic care needs and the use of new technologies.

Finally, the used threshold (≥70%) and the absence of the stability criterion could represent other limitations of the study. Nevertheless, a conventionally agreed threshold does not exist. It depends upon sample numbers, the aim of the study, and resources [25]. About stability, in their analysis of systematic reviews of Delphi techniques from different sectors of the health sciences, Niederberger et al. [26] found that the stability of the judgments did not play a pivotal role in the Delphi articles.

## 5. Conclusions

Since chronic pain patients require high-intensity care, it is appropriate to define the most appropriate strategies helpful during the pandemic. Moreover, it is important to start thinking in terms of post-pandemic scenarios. In this Delphi-based study, the panelists achieved a consensus for all but one of the statements. In particular, the survey underlines the need to make important organizational changes. Diagnostic and therapeutic pathways should be implemented, and the application of new technologies for pain management seems to be a fascinating perspective. Further investigations are needed to identify the appropriate management of post-COVID-19 chronic pain. Finally, remote assistance for chronic pain must be properly regulated.

## Figures and Tables

**Table 1:** First semi-structured questionnaire.

**PREVENTION OF CHRONIC PAIN**
**Regarding the management of acute pain, when the provision of care is reduced:**
It is necessary to establish priority criteria. However, in the context of an emergency, acute pain management must necessarily be maintained or even implemented, regardless of priorities. Even for acute pain, remote assistance strategies must be implemented (e.g., through telemedicine). The management of acute pain must combine approaches that refer to the various specialties involved.
Comments:
**CHRONIC PAIN TREATMENT**
**About difficulties to access pain clinics/ambulatory:**
It is necessary to maintain the appointments of the follow up clinic visits, avoiding dangerous delays. Telemedicine approaches must be activated for follow-up visits. It may be sufficient to maintain periodic telephone (or e-mail) contacts with users, without resorting to more logistically complex methods of telemedicine.
Comments:
**When the availability of diagnostic procedures is limited, it would be appropriate to:**
Concentrate efforts on pharmacological management, delaying instrumental diagnosis if possible. Apply priority criteria for necessary specialist services (e.g., imaging, neurosurgical visits, etc.). Guarantee of an appropriate diagnostic pathway for all patients, since the diagnosis phase is fundamental in the management of chronic pain.
Comments:
**Regarding the prescription of analgesic drugs, it would be appropriate to:**
Provide patients with prescriptions for less than 30 days of therapy, but scheduling closer clinical follow-ups (e.g., via telemedicine). Involve government authorities and multiple professional figures (e.g., general practitioners) to structure pathways aimed at simplifying the processes of prescription and distribution. Use the channel of the direct drug distribution from territorial hospital pharmacies.
Comments:
**When access to non-pharmacological therapies (interventional, surgical, etc.) is limited, it would be appropriate:**
To maintain and enhance only drug therapy and delay interventions over time. Interventional / surgical procedures must have pre-established priority channels. To apply priorities for hospitalizations to carry out analgesic procedures according to shared protocols (e.g., within the pain network).
Comments:
**To avoid improper use of priority criteria for referring chronic pain patients suffering to a specialist visit, it would be advisable:**
The definition of shared pathways to establish criteria of appropriateness for a pain specialist assessment. To create an IT system for verifying the appropriateness. To provide an increase in the training offer for non-specialist doctors in order to avoid incongruous requests.
Comments:
**Regarding the use of telemedicine for the management of chronic pain:**
Telemedicine is a valid approach and must be implemented, but it cannot be used for all patients (e.g., in some age groups). Although optimal, this approach has important limitations including the need to obtain collaboration between the various professional figures involved, identification of suitable evaluation tools, implementation of monitoring instruments, and the ability to assess personal needs and expectations. Telemedicine can allow personalized management of painful problems, but it presents organizational obstacles that are often insurmountable, especially in rural/suburban areas (e.g., connection with the NHS for prescriptive flow and management of reservations).
Comments:
**In the setting of pediatric subjects, chronic pain management should be organized:**
Through pathways that provide a combination of ambulatory accesses and remote assessments based on individual cases. Especially through telemedicine processes. It would be advisable to favor face-to-face assessments.
Comments:
**CONSEQUENCES OF INADEQUATE TREATMENT**
**In the worsening of painful symptoms or deterioration of clinical conditions and quality of life, it is useful:**
Structuring pathways (including multidisciplinary approaches) that can guarantee a rapid re-evaluation and the most suitable therapeutic program. To advise patients to the general practitioner who will request a specialist consultation when necessary. For this purpose, it is necessary to implement the assistance network. To advise patients to access hospitals or other medical institutions to address the pain problem.
Comments:
**In case of new-onset or worsening of psychological/psychiatric problems in patients suffering from chronic pain, it is recommended to:**
Schedule in-person specialist assessments (psychologist/psychiatrist) as soon as possible. Activate a teleconsultation system with the psychologist/psychiatrist. Increase the training and the hospital staffing of qualified personnel (psychologist/psychiatrist expert in pain issues).
Comments:
**When managing the side effects of analgesic therapies is difficult, it is advisable to:**
Schedule more frequent outpatient checkups as side effects need to be corrected as soon as possible. Create a therapeutic alliance with general practitioners to alert the referral pain therapist and avoid continuous hospital access. Inform the patient of possible side effects and provide a tool to monitor them (self-managed by the patient himself).
Comments:
**PERSPECTIVES**
**What are the opportunities for the healthcare system during and after the pandemic?**
To provide additional territorial pain therapy services. To strengthen the entire pain care networks and structure remote assistance pathways. To enhance the role of general practitioners through certified training courses and entrust them with the management of less problematic cases.
Comments:
**Are you in favor of using medical APPs (for example, as a reminder for taking medications) to improve therapeutic adherence?**
Yes, because also as an effect of the pandemic, the digital literacy level of the population has significantly increased. I would try starting from the simplest models, perhaps relying on local task forces that illustrate the technological solutions to be used. It is necessary to assess the specific needs of the patient. In other words, this approach may be useful but not for everyone.
Comments:
**What strategies would you adopt to manage chronic pain problems secondary to COVID-19 (long-COVID)?**
It would be advisable to set up a network (Hub/Spokes) with territorial clinics coordinated by provincial/regional reference centers and managed by pain therapists. It is necessary to organize several territorial multidisciplinary units. The management can be performed by general practitioners who would act as the first filter and direct the most complex cases to other specialists (neurologists, pain specialists, physiatrists).
Comments:
**In patients with chronic problems secondary to the acute phase of COVID-19 disease (e.g., long hospital stays), how rehabilitation programs should be organized?**
It is essential to organize rehabilitation programs managed in a multidisciplinary environment and coordinated by physiatrists (e.g., motor/respiratory rehabilitation). It is necessary to start remote consultation services (e.g., telemedicine) managed by specialists in physiatry. The general practitioner will assess the need for a rehabilitation program, possibly providing for the identification of the most suitable territorial structures.
Comments:
**Based on the healthcare issues during the pandemic, how do you consider the use of data science and artificial intelligence in pain medicine?**
A useful approach to calculate, based on clinical diaries compiled by patients or caregivers, the frequency and priority for the first visit and the frequency of follow-ups. A useful approach but only for research purposes. An opportunity to move towards the virtual pain therapist (e.g., IBM’s Watson path).
Comments:

**Table 2:** Expert panel respondents.

	First Delphi Round	Second Delphi Round
*n* (M/F)	20 (12/8)	20 (11/9)
Age (%):		
30–40	15%	10%
41–50	25%	25%
51–60	35%	30%
>60	25%	35%
Geographic area (Italy) (%):		
North-West	25%	20%
North-East	20%	30%
Center	20%	15%
South and Islands	35%	35%
Disciplines (%):		
Pain therapy	40%	25%
Oncology	15%	15%
General medicine	10%	10%
Rheumatology	5%	0%
Neurology	10%	15%
Psychiatry	5%	5%
Pediatrics	5%	0%
Anesthesiology	5%	15%
Orthopedics/Physiatry	5%	10%
Addiction medicine	0%	5%

**Table 3:** Comparative judgments in the first and second Delphi rounds.

	First Round *	Median (IQR)	Second Round *	Median (IQR)
**ORGANIZATIONAL ISSUES**				
1 The health problems that emerged during the pandemic make it necessary to reshape the organization of chronic pain therapy.	75%	5 (1.3)	90%	4 (1)
2 With regard to the difficulties in accessing the antalgic therapy clinics, it is advisable to schedule close-up follow-up visits to reduce therapeutic delays.	65%	4.5 (2)		
*2.1 (rephrased). In the pandemic and post-pandemic period, despite the difficulties in accessing the antalgic therapy clinics, it is still advisable to schedule close follow-ups.*			90%	4 (1)
3 It is desirable to involve the authorities and multiple professionals (e.g., general practitioners, pediatricians, pharmacists) to set up pathways aimed at simplifying the prescription and the distribution process.	75%	5 (1)	80%	4 (1)
4 It is desirable to define priority criteria for interventional and surgical procedures for pain relief.	95%	5 (0.3)	90%	5 (1)
5 In pediatric patients it is advisable to organize tailored pathways that include outpatient access and remote assessments.	75%	4 (1.3)	80%	4.5 (1)
6 In case of worsening of painful symptoms it is advisable to activate the territorial assistance network through priority access to the general practitioner.	75%	4 (1.3)	75%	4 (1.3)
7 The general practitioner must act as the first filter and subsequently refer complex patients to the various specialists according to well-defined multidisciplinary pathways.	90%	5 (0.3)	95%	5 (1)
8 In the multidisciplinary approach of the rapidly worsening chronic patient, it is appropriate to consider activating the priority consultation with the psychologist/psychiatrist.	75%	4 (1.3)	80%	4 (1)
**DIAGNOSIS AND THERAPY**				
1 When the availability of diagnostic procedures is limited, patients should be selected according to priority criteria.	90%	5 (1)	80%	4 (1)
2 When the availability of diagnostic procedures is limited, efforts need to be focused on pharmacological management.	45%	3 (1)		
*2.1 (Rephrased) While waiting to complete the instrumental diagnosis, it is advisable to start a pharmacological process.*			95%	4 (1)
3 Regarding the prescription of analgesic drugs, it is conceivable to provide patients with prescriptions for less than 30 days of therapy only by scheduling closer clinical checks.	65%	4 (1)		
*3.1 (Rephrased) If closer checks cannot be scheduled, it is advisable to prescribe analgesic therapy for up to 30 days.*			70%	4 (2)
4 In case of reduced access to interventional or surgical therapies, it is advisable to maintain and, if necessary, enhance the drug therapy.	65%	4 (1.3)		
*4.1 (Rephrased) While waiting to be able to carry out planned interventional or surgical therapies, it is advisable to maintain or enhance the drug therapy.*			90%	4 (1)
5 In case of difficult or complex pain, it is a priority to apply protocols coordinated by the pain therapist that include multimodal therapy.	90%	5 (1)	70%	4.5 (2)
6 In patients with post-COVID-19 chronic pain it is important to provide specific therapeutic pathways.	65%	4 (2)		
*6.1 (Rephrased) In patients with post-COVID-19 chronic pain it would be desirable to provide specific therapeutic pathways in dedicated clinics.*			**55%** ^†^	4 (2.3)
**TELEMEDICINE AND FUTURE PERSPECTIVES**				
1 On the basis of the healthcare problems during the pandemic, the use of new technologies is useful.	85%	4 (1)	90%	4.5 (1)
2 During and after the pandemic, the use of telemedicine and remote clinical and instrumental monitoring improves the management of chronic pain.	75%	4 (1.3)	85%	4.5 (1)
3 In situations of reduced availability of hours of analgesic therapy, it is advisable to implement remote care strategies for the management of primary and secondary chronic pain (including cancer pain).	70%	4 (2)	**65%**	4 (2)
4 The remote management of chronic diseases can improve access to care. Nevertheless, it is desirable that at least the first assessment should be performed in person.	85%	4 (0.3)	80%	4 (1)
5 Telemedicine must be considered a tool that integrates clinical practice.	85%	4.5 (1)	95%	4.5 (1)
6 To improve therapeutic adherence, the development of new scientifically validated technological aids (e.g., Apps, wearable devices, remote control of physiological parameters) is desirable.	80%	5 (1)	90%	5 (1)
7 It is desirable to develop training programs aimed at healthcare professionals, patients, and caregivers for the correct use of new generation digital tools.	95%	5 (0.3)	100%	5 (1)

* Agreement ≥ 4 (Agree or Strongly agree); ^†^ A score ≥ 3 was obtained in 75% of panelists. Bold serves to highlight the values below the threshold

## Data Availability

The data presented in this study are available on request from the corresponding author.
